# Scientific Rationale Supporting the Clinical Development Strategy for the Investigational Aurora A Kinase Inhibitor Alisertib in Cancer

**DOI:** 10.3389/fonc.2015.00189

**Published:** 2015-08-24

**Authors:** Huifeng Niu, Mark Manfredi, Jeffrey A. Ecsedy

**Affiliations:** ^1^Department of Translational Medicine, Takeda Pharmaceuticals International Co, Cambridge, MA, USA; ^2^Department of Oncology Biology, Takeda Pharmaceuticals International Co, Cambridge, MA, USA

**Keywords:** Aurora, combination therapy, biomarkers, alisertib, mitosis

## Abstract

Alisertib (MLN8237) is a selective small molecule inhibitor of Aurora A kinase that is being developed in multiple cancer indications as a single agent and in combination with other therapies. A significant amount of research has elucidated a role for Aurora A in orchestrating numerous activities of cells transiting through mitosis and has begun to shed light on potential non-mitotic roles for Aurora A as well. These biological insights laid the foundation for multiple clinical trials evaluating the antitumor activity of alisertib in both solid cancers and heme-lymphatic malignancies. Several key facets of Aurora A biology as well as empirical data collected in experimental systems and early clinical trials have directed the development of alisertib toward certain cancer types, including neuroblastoma, small cell lung cancer, neuroendocrine prostate cancer, atypical teratoid/rhabdoid tumors, and breast cancer among others. In addition, these scientific insights provided the rationale for combining alisertib with other therapies, including microtubule perturbing agents, such as taxanes, EGFR inhibitors, hormonal therapies, platinums, and HDAC inhibitors among others. Here, we link the key aspects of the current clinical development of alisertib to the originating scientific rationale and provide an overview of the alisertib clinical experience to date.

## Alisertib: A Highly Selective Aurora A Kinase Inhibitor

Early interest in targeting Aurora A for cancer treatment stemmed in part from the fact that the gene, localized to chromosome 20q13.2, is commonly amplified and overexpressed in a diversity of cancer types (1–7). Aurora A amplification and overexpression is correlated to a worsened prognosis for patients. For example, a meta-analysis study of 5523 cancer patients from thirty-nine studies demonstrated that patients with higher Aurora A expression levels had a significantly worsened survival outcome irrespective of disease type or stage (8). Aurora A overexpression is also thought to drive oncogenesis by causing genomic instability; this proposal is supported by evidence demonstrating that Aurora A overexpression transforms normal cells into cancer cells in experimental studies (7, 9–13). As such, Aurora A has been considered an attractive target for treating cancer and multiple Aurora kinase inhibitors have been developed and tested in cancer patients, including alisertib (MLN8237).

**Figure 1:**
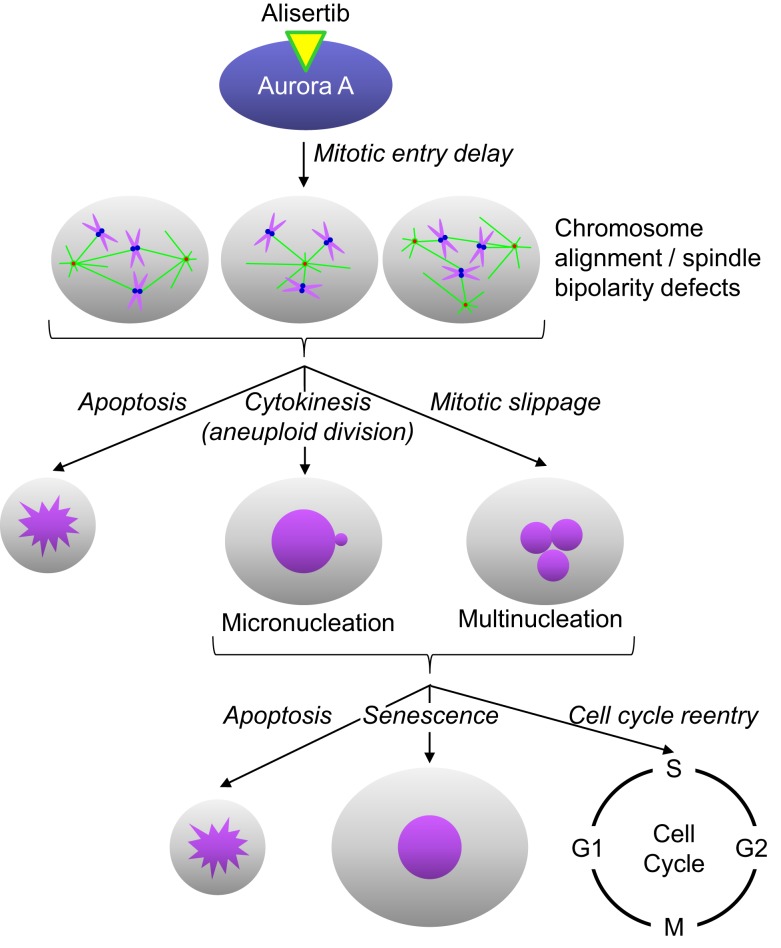
**Alisertib mechanism of action**. Alisertib selectively binds to and inhibits Aurora A kinase in cells. Inhibition of Aurora A results in delayed mitotic entry and progression through mitosis leading to an accumulation of cells with a tetraploid DNA content. Mitotic cells treated with alisertib display a variety of defects, including monopolar, bipolar, and multipolar spindles, all with misaligned chromosomes. These cells can die directly out of mitosis via apoptosis, undergo aneuploid cytokinesis or exit mitosis without undergoing cytokenesis through a process known as mitotic slippage. The resulting interphase cells typically display gross nuclear defects including micronucleation and multinucleation. These cells will then undergo apoptosis, senescence or reenter the cell cycle; the specific fate is likely dictated by the extent of DNA damage/aneuploidy that occurred in any cells as a result of the abnormal mitotic division as well as other underlying genetic factors.

Alisertib is a benzazepine containing small molecule inhibitor of Aurora A (14). In enzymatic, cell and *in vivo* assays, alisertib has proven to selectively inhibit Aurora A (14). For example, alisertib demonstrated selectivity for Aurora A relative to other kinases in an *in vitro* screen of 205 kinases, and was >200-fold more potent against Aurora A than the structurally related kinase Aurora B in cellular assays. The selectivity for Aurora A was substantiated by mechanism of action studies in cultured cancer cells and tumors grown as xenografts in immunocompromised mice. Alisertib concentrations that lead to cell cycle arrest and death are preceded by phenotypic changes consistent with Aurora A inhibition; including increased incidence of tetraploid (4N) cells as well as mitotic cells with abnormal mitotic spindles and misaligned chromosomes (Figure 1). Furthermore, alisertib did not affect the viability of cancer cell lines expressing a drug-resistant Aurora A mutation, suggesting that its antitumor activity occurs predominantly through Aurora A inhibition (15).

Alisertib has demonstrated antitumor activity across a broad array of solid cancers and heme-lymphatic experimental tumor models when grown *in vitro* and *in vivo* (14, 16–24). In addition, single-agent alisertib has been evaluated in multiple clinical trials and has shown clinical activity across a diversity of cancer types, including solid and hematological cancers in adult and pediatric populations. Though alisertib displays differential antitumor activity across experimental tumor models and in cancer patients, the biological underpinnings for alisertib sensitivity remain unclear. Multiple hypotheses have been developed based on Aurora A biology and data collected in experimental models that predict which cancers will most likely respond to alisertib as a single agent or in combination with other therapeutic agents. In this review, the data supporting some of these concepts is shared.

## Early Clinical Studies for Dose/Schedule Selection and Proof of Mechanism

Alisertib has been formulated for oral administration in patients and is available as an enteric-coated tablet and as a liquid solution for pediatric cancers. In two phase 1 studies of alisertib in adults with advanced solid malignancies (25, 26), and in one phase 1 study of alisertib in adults with hematological cancers (27), the single agent maximum tolerated dose was determined to be 50 mg dosed orally twice daily for 7 days followed by 14 days of non-treatment. This dose was selected for further single-agent alisertib evaluation in additional clinical trials of adult cancer patients. Alisertib was also evaluated once daily for 21 days followed by 14 days of non-treatment; 50 mg was the maximum tolerated dose on this schedule (25, 26). The most common dose limiting toxicities (DLTs) observed with alisertib were fatigue, nausea, neutropenia, and stomatitis. These toxicities reflect the pharmacologic activity of alisertib as a cell cycle inhibitor in highly proliferative tissues. Other common alisertib-associated toxicities included alopecia, anorexia, leukopenia, anemia, thrombocytopenia, asthenia, vomiting, diarrhea, and somnolence. The occurrence of somnolence was likely due to the benzodiazepine-like structure of alisertib.

Alisertib has also been evaluated in pediatric cancer patients. This was in part based on the observation that alisertib was active against a range of pediatric tumors grown *in vitro* and *in vivo*, in particular, neuroblastoma and acute lymphocytic leukemia (28, 29). In a phase 1 study of children with solid tumors, the maximum tolerated dose of alisertib in children with solid tumors was 80 mg/m^2^ administered orally once daily for 7 days followed by 14 days of non-treatment (30). The exposures achieved with this dose is approximately 1.5-fold greater than the adult maximum tolerated dose of 50 mg twice daily. Mucositis/stomatitis, mood alteration/depression, neutropenia, and elevated alkaline phosphatase were the DLTs in these patients; neutropenia being the most frequently occurring dose-limiting toxicity. In addition to depression, other mood alterations included impaired memory, agitation, euphoria, and somnolence, predominantly grade 1 and 2. Hand–foot–skin reactions were also observed in these patients.

The selectivity of alisertib for Aurora A relative to Aurora B observed in non-clinical experimental models also translated into the cancer patients. Pharmacodynamic studies performed on tumor biopsies obtained from patients prior to and after alisertib dosing demonstrated an exposure-related decreases in tumor mitotic cells with aligned chromosomes and bipolar spindles in the post-dose samples; phenotypes consistent with Aurora A inhibition (25, 31). Moreover, skin and tumor biopsies taken prior to and after alisertib dosing had increased in mitotic cells in the post-treatment biopsies with serine 10 phosphorylated Histone H3. As serine 10 phosphorylation of histone H3 is catalyzed by Aurora B in cells, these data demonstrate that alisertib does not significantly inhibit Aurora B at the single agent maximum tolerated dose (25, 26, 31). Confirmation of alisertib’s functional selectivity for Aurora A in cancer patients allows for its rational development for treating multiple types of cancers as single agent or in combination with other therapeutic agents.

Population-based pharmacokinetic–pharmacodynamic modeling demonstrate that alisertib steady-state exposures achieved with 50 mg twice daily for 7 days is associated with pharmacodynamic activity in tumors and a low probability for DLTs (31). Moreover, patients with intolerable treatment related toxicities at 50 mg twice daily can be dose reduced to 40 or 30 mg on the same schedule and still maintain tumor pharmacodynamic effects. Overall, multiple tolerated and pharmacodynamically active dose/schedules have been identified in adult and pediatric patients allowing for sufficient flexibility in alisertib dosing that can be taken advantage of for single-agent evaluation and for combining with multiple other therapeutic agents.

## Alisertib Single-Agent Rationale

### Neuroblastoma

**Figure 2:**
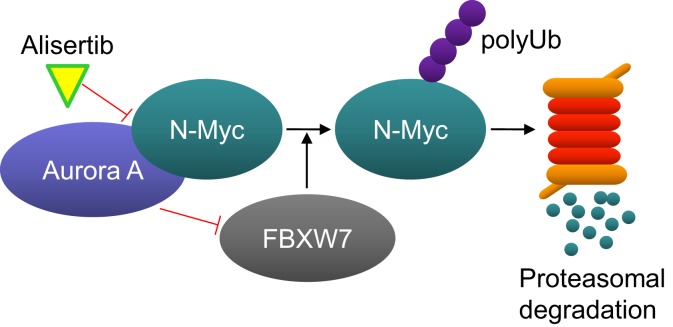
**Aurora A protects N-Myc from proteasome-mediated degradation**. Aurora A binds to N-Myc, thereby preventing it from being ubiquitinated by the E3-ligase FBXW7. Alisertib binds to Aurora A and changes its confirmation in a manner that disrupts its interaction with N-Myc. N-Myc is than ubiquitinated by FBXW7 and subsequently degraded by the proteasome.

Interest for developing alisertib in neuroblastoma initially derived from an evaluation of alisertib antitumor activity in a large set of pediatric cancer models executed by the Pediatric Preclinical Testing Program which maintains the mission for identifying novel therapies for treating childhood cancers. Alisertib potently inhibited the growth of neuroblastoma cells *in vitro* and resulted in maintained complete responses in three of seven neuroblastoma xenograft models grown in immunocompromised mice; responses which surpassed the activity of other agents tested in these models (29). Subsequent to these findings it was proposed that Aurora A is essential for the growth and survival of *MYCN*-amplified neuroblastoma cells. Aurora A binds to and stabilizes N-MYC by protecting it from FBXW7 E3 ubiquitin ligase-mediated proteasomal degradation in a manner that is independent from Aurora A catalytic activity (Figure 2) (32). Furthermore, alisertib and the structurally related molecule MLN8054 bind to Aurora A’s catalytic domain in manner that causes an allosteric shift in the protein thereby disrupting its’ interaction with N-Myc (33, 34). Interestingly, the allosteric shift at the Aurora A/N-Myc interaction site caused by alisertib does not occur with all catalytic inhibitors of Aurora A kinase. Several studies have also demonstrated antitumor activity of Aurora A inhibition in *MYCN*-amplified neuroblastoma models. For example, treatment of TH-*MYCN* neuroblastoma mice with MLN8054 resulted in decreased N-Myc protein expression, diminished expression of N-Myc target genes, tumor regressions and increased survival (33). Other Aurora A inhibitors also decreased N-Myc expression resulting in inhibited tumor growth of other *MYCN*-amplified tumors (34, 35).

As a result of these findings, the Children’s Oncology Group led a phase 1 study of single-agent alisertib in children with relapsed/recurrent solid tumors including neuroblastoma to determine the maximum tolerated dose, safety profile and pharmacokinetics of alisertib. In this study, 4 out of 11 evaluable neuroblastoma patients treated with alisertib had stable disease (≥6 cycles) (30). As described above, the DLTs in these patients was mucositis, neutropenia, and mood alteration. A phase 2 study of alisertib in young patients with recurrent or refractory solid tumors or leukemias including neuroblastoma has also recently been completed (NCT01154816). Currently, there is an ongoing study being led by the New Approaches to Neuroblastoma Therapy (NANT) consortium in recurrent or resistant neuroblastoma patients combining alisertib with the FDA-approved drugs for neuroblastoma treatment, irinotecan and temozolomide (NCT01601535). In this study, there is a plan to compare *MYCN* status to patient outcome.

### Small cell lung cancer

Similar to neuroblastoma, SCLC has an etiological link to Myc-family of oncogenes including *MYC* (c-Myc), *MYCN* (N-Myc) and *MYCL1* (L-Myc). Amplification and overexpression of these genes is thought to constitute 18–31% of SCLCs (36–38). Multiple preclinical studies have suggested that SCLCs with Myc activation or amplification are notably sensitive to Aurora kinase inhibitors. For example, SCLC cell lines with *MYC*, *MYCN*, and *MYCL1* activation or amplification were the most sensitive in a viability screen of 87 cell lines using the dual Aurora A and Aurora B kinase inhibitor PF-03814735 (39). In a separate screen of 34 SCLC cell lines, four structurally diverse Aurora kinase inhibitors VX680, alisertib, PHA680632, and ZM447439 were most effective against the *MYC*-amplified cell lines (37). Studies with the dual Aurora A and Aurora B kinase inhibitor VX680 demonstrated that it selectively killed human retinal pigment epithelial cells that overexpress c-Myc (40).

In a phase 2 study of single-agent alisertib in five types of advanced refractory or relapsed solid cancers, encouraging activity was seen in SCLC (41). Objective partial responses were observed in 10 of the 48 (21%) SCLC enrolled in this study; these responses occurred in both chemotherapy-sensitive and chemotherapy-resistant disease, the latter which has a worse prognosis. The most common grade 3–4 adverse events in the SCLC patients from this phase 2 study were neutropenia, anemia, leucopenia, and thrombocytopenia, which are consistent with those noted in earlier trials of alisertib. Currently, a phase 2 study of alisertib in combination with paclitaxel compared to placebo in combination with paclitaxel in patients with second line relapsed or refractory SCLC is ongoing (NCT02038647).

### Neuroendocrine prostate cancer

Neuroendocrine prostate cancer is thought to evolve from late stage prostate adenocarcinoma concurrent to become resistant to hormonal therapy (42, 43). As part of that transition, neuroendocrine prostate cancers become more genomically unstable than prostate adenocarcinoma and include co-amplification of MYCN and Aurora A (44, 45). Given this observation, the relative sensitivity of several prostate adenocarcinoma and neuroendocrine cancer models to the pan-Aurora inhibitor danusertib was tested (44). In a viability screen of four cell lines grown in cell culture, the one neuroendocrine prostate cancer model was significantly more sensitive to danusertib than the three adenocarcinoma cell lines. Danusertib also displayed greater antitumor activity in LNCaP cells transfected with MYCN than vector–control LNCaP cells and was more effective in inhibiting the growth *in vivo* of a neuroendocrine prostate cancer model relative to an adenocarcinoma model. As a result of these observations, a phase 2 in NEPC is ongoing with single-agent alisertib (NCT01799278).

### Atypical teratoid/rhabdoid tumors

Aurora A is a promising target for therapy in ATRT and alisertib has demonstrated to be a potent radiosensitizer in ATRT experimental models (46). ATRT is a rare and highly malignant central nervous system (CNS) tumor usually diagnosed in childhood. ATRT represents around 3% of CNS pediatric cancers and has a high mortality rate with a very poor prognosis. Mutation or deletion of the tumor suppressor gene INI1/hSNF5 occurs in the majority of ATRTs. hSNF5/INI1 is a component of the chromatin remodeling SWI/SNF complex which regulates many proteins involved in chromatin structure. Aurora A is a direct downstream target of hSNF5/INI1. hSNF5/INI1 acts to repress Aurora A expression; as such, loss of INI1/hSNF5 in rhabdoid tumors leads to aberrant overexpression of Aurora A which is required for tumor survival in non-clinical cancer models (47). These preclinical findings supported the use of alisertib for ATRT patients. Wetmore et al. reported an encouraging result for clinical use of alisertib as single agent in recurrent ATRT in four children (48). Patients with recurrent or progressive ATRT received oral administration of alisertib 80 mg/m^2^ once daily for 7 days of a 21-day treatment cycle. Disease burden was evaluated by brain and spine MRI and by evaluation of spinal fluid cytology (lumbar puncture) after two cycles of alisertib and every 2–3 cycles thereafter for as long as the patients remained free from tumor progression. All four patients had disease stabilization and/or regression after three cycles of alisertib therapy. Two patients on therapy showed stable disease regression for 1 and 2 years. Consistent with other pediatric studies, alisertib in these patients had moderate but manageable toxicities, including neutropenia, leukopenia, thrombocytopenia, anemia, somnolence, and alopecia. Alisertib appears a promising therapeutic agent in this pediatric population. A phase 2 study is ongoing to further evaluate alisertib in the treatment of children with ATRT.

### Breast cancer

**Figure 3:**
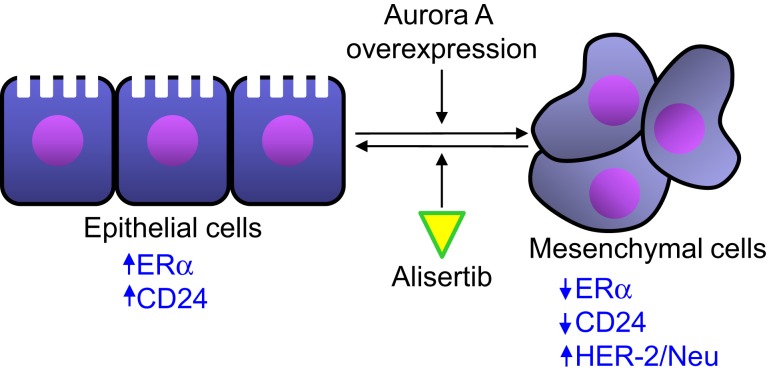
**Aurora A activity affects epithelial to mesenchymal transition in breast cancer cells**. Aurora A overexpression leads to the transition of breast cancer cell from an epithelial phenotype to a mesenchymal phenotype, leading to decreased ERα and CD24 expression and HER-2/Neu overexpression. Alisertib counteracts the effects of Aurora A overexpression leading to an epithelial to mesenchymal transition reversion.

Single-agent alisertib efficacy was evaluated in a phase 2 study that comprised five advanced solid tumor indications including breast cancer (41). Among response-evaluable breast cancer patients, objective response (all partial responses) was observed in 9 [18%, 95% confidence interval (CI) = 9–32%] of 49 women with breast cancer. The most common grade 3–4 adverse events in the breast cancer patients from this study included neutropenia, fatigue, leucopenia, and stomatitis. The antitumor activity of alisertib was particularly encouraging in the hormone receptor-positive and HER2-negative subgroups. Median progression-free survival in this subgroup was 7.9 months (95% CI 4.2–12.2). This clinical finding is supported by previously reported preclinical results. D’Assoro et al. demonstrated that Aurora A drives the transition of estrogen receptor α-positive (ERα^+^) breast cancer cells from an epithelial to a highly invasive mesenchymal phenotype (49). The transition from an ­epithelial-like to a mesenchymal-like phenotype was characterized by reduced expression of ERα, HER-2/Neu overexpression and loss of CD24 surface receptor (CD24^–/low^) and overexpression of Aurora A (Figure 3). Aurora A overexpression induces epithelial–mesenchymal transition (EMT) and a cancer stem cell-like phenotype. Inhibition of Aurora A by alisertib *in vitro* reverses EMT and suppresses the self-renewal ability of CD24^–/low^ breast cancer. Moreover, molecular targeting of Aurora A by shRNA *in vivo* restores a CD24^+^ epithelial phenotype and inhibits the development of distant metastases. Other studies demonstrated that increased Aurora A activity may result in anti-hormonal therapy resistance in breast cancer (50). Aurora A induces endocrine resistance through down-regulation of ERα expression in initially ERα^+^ breast cancer cells (51). In breast cancer patients, high Aurora A expression is associated with poor survival particularly in node-negative ER-positive breast cancer patients (50). Taken together, alisertib could be a novel promising therapeutic agent to selectively eliminate highly invasive cancer cells and improve the disease-free and overall survival of ER-positive breast cancer patients resistant to conventional endocrine therapy.

## Alisertib Combination Development Rationale

### Taxanes

A considerable amount of data has accumulated in preclinical studies suggesting the benefit of combining Aurora kinase inhibitors with antimicrotubule perturbing agents. This class of anticancer therapies which comprises the taxanes, vinka alkaloids, and the epothilones is among the most commonly used for treating both solid and hematological cancers. Multiple preclinical studies have demonstrated the beneficial combination of inhibiting Aurora kinase with this class of agents (52–60). For example, alisertib combined with the taxanes paclitaxel and docetaxel in triple-negative breast cancer tumors grown as xenografts in immunocompromised mice led to additive or synergistic antitumor activity with prolonged tumor growth delay and in some cases durable complete responses after discontinuing treatment (53). Though the underlying biological underpinnings explaining the beneficial combination between antimicrotubule agents remains uncertain, it has been shown that Aurora A inhibition using MLN8054 or RNA interference in the presence of paclitaxel caused cells to rapidly exit mitosis without completing cytokinesis, presumably due to a disruption of the spindle assembly checkpoint (61).

Alisertib administered as a single agent was evaluated in patients with platinum-resistant or -refractory epithelial ovarian, fallopian tube, or primary peritoneal carcinoma (62). Though active in these diseases as a single agent (overall response rate of 10%, durable for 6.9–11.1 months), the activity was not considered sufficient for further development in ovarian cancer as a single agent. Therefore, alisertib was tested in combination with paclitaxel in relapsed and refractory ovarian cancer (NCT01091428). During the phase 1b portion of this study weekly paclitaxel (QWx3) at 80 mg/m^2^ and 60 mg/m^2^ was administered with alisertib dosed twice daily on a 3 days on, 4 days off schedule for three consecutive weeks over 28-day cycles (63). Exposure efficacy modeling was used for selecting the phase 2 dose for this study (53). In addition, alisertib and paclitaxel are being tested in metastatic or locally recurrent breast cancer (NCT02187991) and SCLC (NCT02038647). Numerous other studies have been completed or are ongoing testing alisertib in combination with other microtubule perturbing agents, including Abraxane (nab-paclitaxel) in patients with advanced solid cancers (NCT01677559), docetaxel in patients with advanced solid tumors (NCT01094288), and vincristine and rituximab in patients with relapsed or refractory B-Cell lymphomas (NCT01397825).

### EGFR inhibitors

Epidermal growth factor receptor (EGFR)-targeting antibodies or small molecular EGFR inhibitors are widely used to treat patients with gastrointestinal (GI), breast, head and neck, and lung cancers. However, the clinical efficacy of these agents is limited by intrinsic and acquired resistance factors. Astsaturov and colleagues employed a synthetic lethal screening method and identified Aurora A as a promising hit necessary for cells to survive in the presence of an EGFR inhibitor (64). In addition, they observed synergistic activity of combined inhibition of the EGFR and Aurora A pathways in cancer cells. Combination of erlotinib and alisertib showed synergistic antitumor activity *in vitro* and *in vivo* in lung cancer models (65). Furthermore, Aurora A and EGFR protein expression were assessed by immunohistochemistry in patients with squamous cell cancer of the head and neck (SCCHN) (*n* = 180). Co-expression of elevated levels of Aurora A and EGFR was a poor prognostic factor in SCCHN (66). Recently, Crystal and colleagues established patient-derived resistant NSCLC models to identify effective drug combinations (67). Aurora kinase inhibitors were active in combination with EGFR inhibition in a number of EGFR-mutant cell lines. These data together suggest a potential benefit of such combination therapy in patients. Currently, there is an ongoing phase 1 study evaluating the safety and tolerability of combining alisertib with erlotinib in patients with non-SCLC (NCT01471964).

### Hormonal therapy in breast cancer

A number of evidences suggest alisertib may be a rationale combination partner for hormonal therapy. First, promising alisertib single-agent activity was observed in ER-positive and HER2-negative patients as described above (41); second, Aurora A plays a role in the development of endocrine resistance through activation of SMAD5 nuclear signaling and down-regulation of ERα expression in initially ERα^+^ breast cancer cells (51); and third, aromatase inhibitors (AIs) are used for treatment of ER-positive breast cancer though resistance to AI is a major obstacle to optimal patient outcome. Aurora A is upregulated in AI-resistant cell lines and knockdown studies of Aurora A have shown that it is essential for AI-resistant cell growth. In AI-resistant cell lines, alisertib blocked cell cycle progression at the G2/M phase, interfered with chromosome alignment and spindle pole formation, and preferentially inhibited AI-resistant cell growth compared with parental control cells (68). Furthermore, combination of Aurora inhibitors (alisertib, JNJ-7706621, or danusertib) with fulvestrant is superior to treatment with either of the compounds alone, particularly in AI-resistant cell lines (68). Importantly, this combination may have minimal overlapping toxicities in breast cancer patients. A phase 1/2 trial of alisertib in combination with fulvestrant in patients with hormone receptor-positive metastatic or locally advanced breast cancer is ongoing (NCT02219789).

### Platinums

Platinum-based drugs continue to be the mainstay of therapy for many cancers, such as ovarian and lung cancers; however, chemoresistance (intrinsic or acquired) is a major limitation for platinums as it is for other therapies. Increasing evidence suggests a role of Aurora A in platinum resistance. Elevated expression of Aurora A is associated with poor prognosis in epithelial ovarian cancer patients (69) and high Aurora A expression is correlated with cisplatin-based chemotherapeutic resistance and predicts poor patient overall survival (OS) and progression-free survival in NSCLC (70). Moreover, forced expression of Aurora A increased the resistance of the lung cancer cells to cisplatin and knocked down of Aurora A expression in the cisplatin resistant cells by siRNA resulted in a significantly enhanced sensitivity to cisplatin (70). In addition, combination of alisertib and cisplatin resulted in enhanced antitumor activity *in vivo* in multiple preclinical models (21). In a recent phase 2 clinical trial, alisertib exhibits encouraging single-agent activity in SCLCs, particularly in refractory or chemotherapy-resistant/relapsed patients as described above. Three of twelve patients with refractory or chemotherapy-resistant disease had objective responses to alisertib (41). In earlier studies, alisertib also showed modest single-agent antitumor activity in patients with platinum-resistant ovarian cancers (62). Combination of alisertib with platinums may be a viable strategy for the treatment of patients with platinum-resistant recurrent SCLC and ovarian cancers.

### HDAC inhibitors

Alisertib has shown promising single-agent antitumor efficacy in a phase 2 trial for the treatment of various hematological malignances (71). The overall response rate was 27% (10% CRs) including 100% (1/1) in Burkitt lymphoma (BL), 29% (6/21) in diffuse large B cell lymphoma (DLBCL), and 50% (4/8) in peripheral T-cell lymphoma (PTCL). Recent data from a phase 2 study of alisertib in PTCL led by the South West Oncology Group (SWOG) showed two complete responses and seven partial responses and a response rate (ORR) of 24%. Among the most common subtypes (PTCL NOS, AITL, and ALCL), the ORR was 33% (72). Similar to previously described data with alisertib, myelosuppression was a common adverse effect and constituted the predominant toxicity requiring dose reduction. Mucositis, anorexia, and diarrhea occurred in less than one-quarter of patients and were largely grade 1 or 2 in severity. Grade 1 or 2 fatigue was also common, being observed in nearly half of patients. Nonetheless, two responding patients in this trial received alisertib for 1 year. On the basis of these results, a global phase 3, randomized registration-enabling trial (NCT01482962) was initiated comparing alisertib with investigator’s choice (gemcitabine, pralatrexate, or romidepsin) in patients with relapsed/refractory PTCL. This study was discontinued as a pre-specified interim analysis indicated that the study was unlikely to meet the primary endpoint of superior progression-free survival (PFS) over the standard of care in this treatment setting, although single-agent activity of alisertib was confirmed. In this phase 3 study, alisertib showed a similar ORR compared to the control arm.

The histone deacetylase (HDAC) inhibitors (vorinostat and romidepsin) were approved in the United States for the treatment of cutaneous T-cell lymphoma and romidepsin for the treatment of PTCL. Preclinical data support combining Aurora A inhibitors with HDAC inhibitors. For example, several studies demonstrated that HDAC inhibitors reduce Aurora A expression leading to arrest in the G2/M portion of the cell cycle, abnormal mitotic spindles and followed by apoptosis (73–75). The pan-Aurora kinase inhibitor MK-0457 in combination with the vorinostat enhanced lymphoma cell death through repression of c-Myc and c-Myc responsive micro RNAs (76). Alisertib also demonstrated synergistic antitumor activity when combined with romidepsin in experimental models of T-cell lymphoma (77). Alisertib selectively synergizes with romidepsin by inducing cytokinesis failure in T-cell lymphoma. Cytokinesis failure was confirmed after a corresponding post-treatment increase in CENP-A protein levels. CENP-A is a chromatin-associated protein and plays a role in the final stages of cytokinesis. Overall, these collective data provide a rationale for evaluating alisertib in combination with romidepsin in patients with multiple lymphoma subtypes. A phase 1 trial of alisertib plus romidepsin for relapsed/refractory aggressive B- and T-cell lymphoma is ongoing (NCT01897012).

## Summary

To date, many clinical studies have been conducted to evaluate antitumor efficacy of alisertib in patients with diverse solid tumors or hematologic malignancies. Treatment related adverse events (in ≥10% of patients) of single-agent alisertib are summarized in Table 1 (25, 26, 41). Although alisertib has shown single-agent clinical activity in multiple tumor settings, identification of appropriate combination partners and sensitive patient populations is required to ensure that an acceptable risk/benefit profile can be achieved. Aurora A has been implicated in the development of resistance to multiple chemotherapies and targeted agents and preclinical data suggest that alisertib can be combined with multiple therapies to yield additive or synergistic antitumor activity. Furthermore, combinations with targeted therapies might yield more favorable clinical risk/benefit profile than combinations with chemotherapeutic partners due to decreased risk for overlapping toxicities. Lastly, identification of potential predictive biomarkers for alisertib will significantly increase the likelihood of expanding the clinical risk/benefit profile. As such, many correlative studies are ongoing to identify predictive biomarkers which could lead to a precision medicine strategy for alisertib.

**Table 1:** **Most common treatment-emergent adverse events of alisertib dosed at 50 mg orally twice daily for 7 days followed by 14 days of non-treatment**.

	All grades^a^	Grade **≥**3^b^
Gastrointestinal disorders	Diarrhea, nausea, stomatitis, vomiting, abdominal pain, constipation	Stomatitis, diarrhea
Blood and lymphatic system disorders	Neutropenia, anemia, thrombocytopenia, leukopenia, febrile neutropenia	Neutropenia, anemia, thrombocytopenia, leukopenia, febrile neutropenia
General disorders and administration site conditions	Fatigue, pyrexia, asthenia, edema peripheral	Fatigue
Skin and subcutaneous tissue disorders	Alopecia	
Nervous system disorders	Somnolence, headache, dizziness	
Metabolism and nutrition disorders	Decreased appetite, dehydration	
Respiratory, thoracic and mediastinal disorders	Dyspnea, cough	

*^a^Treatment-emergent adverse events of alisertib in ≥10% patients*.

*^b^Treatment-emergent grade 3–4 drug-related adverse events in ≥5% patients*.

## Conflict of Interest Statement

The authors declare that the research was conducted in the absence of any commercial or financial relationships that could be construed as a potential conflict of interest.
